# 

**Published:** 1888-02

**Authors:** 


RECEIPTED—1887—Drs. J. T. Hunter, M. M. Crowder, T. E. Morris, W. E. Quinn, C. J. Carter, A. H. Askew, W. H. Stewart, T. H. Ackerman, D· D. Maxwell, W. P. Sheley, to April; D. O. McGehee, H. T. Webb, A. J· Davis, C. M. Kintz J. E. Pope, J. D. Jackson, J. H. Duggan, L. Alison, T. Bailey, T. Beaty.

For 1888—Jno. A. Gordon, to July; Z. J. Scott, T. M. Palmer, R. M. McBee, to Ju.y; J. w. Bennett, D. F. Wilkins, A. W. Calhoun, A. D. Coleman, to May; J. E. Trippe, W. J. McAlpine, J. M, Stansell. W. M. Fowlkes, T. S. Parham, H. Perdue, A. A. Latta, P. C. Tircuit, R. J. Talbert, J. R. Johnson, C. C. Hart, J. M. Guess, to June; J. C. Beauchamp, John B. Foster, H. Allson, to July; M. T. Bell, R. B. McCants. M. T. Bell, S. W. Eaton.

SANDERS & SONS' EUCALYPTI EXTRACT (*Eucalyptol*).—Apply to Dr. Sanders, Dillon, Iowa, for gratis supplied reports on cures effected at the clincs of the Universities of Bonn and Griefswald.

**Sharp & Dohme,** of Baltimore, prepare most beautiful and reliable preparations. Their hypodermic tablets, their gelatine and sugar-coated pills and granules, their solid and fluid extracts, syrups, lozenges, trituates, and indeed all their numerous preparations, are excellent and reliable, See their advertisement on second cover page.

